# Screening and surveillance of multiple solid tumours using plasma placental-like chondroitin sulfate A (pl-CSA)

**DOI:** 10.7150/ijms.39444

**Published:** 2020-01-01

**Authors:** Juzuo Zhang, Beini Sun, Kang Zhang, Zhilong Chen, Wenhan Yang, Guodong Wu, Li Tian, Zhonglin Xiao, Baozhen Zhang, Shiling Chen, Aiwen Le, Youhui Qian, Shaowu Ye, Rihong Zhai, Xiujun Fan

**Affiliations:** 1 Center for Reproduction and Health Development, Shenzhen Institutes of Advanced Technology, Chinese Academy of Science, Shenzhen, 518055, China.; 2 Carson Cancer Center, Guangdong Key Laboratory for Genome Stability & Disease Prevention, Shenzhen University School of Medicine, Shenzhen, 518055, China.; 3 College of Biological and Food Engineering, Huaihua University, "Double First-Class" Applied Characteristic Discipline of Bioengineering in Hunan High Educational Institution, Huaihua, 418000, China.; 4 Department of Obstetrics and Gynaecology, the Sixth Affiliated Hospital of Shenzhen University School of Medicine, Shenzhen, 518052, China.; 5 Department of Gynecology and Obstetrics, Nanfang Hospital, Southern Medical University, Guangzhou, 510515, China.; 6 Department of Oncology, Wuzhou People's Hospital, Wuzhou, 54300, China.; 7 Department of Thoracic Surgery, the First Affiliated Hospital of Shenzhen University School of Medicine, Shenzhen, 518055, China.; 8 School of Life Science, Heilongjiang University, Harbin, 150080, China.; 9 College of Veterinary Medicine, Hunan Agricultural University, Changsha, 410128, China.

**Keywords:** circulating pl-CSA, biomarker, cancer screening

## Abstract

**Rationale:** Placental-like chondroitin sulfate A (pl-CSA) is known to be exclusively synthesized in multiple cancer tissues and associated with disease severity. Here, we aimed to assess whether pl-CSA is released into bio-fluids and can serve as a cancer biomarker.

**Methods:** A novel ELISA was developed to analyse pl-CSA content in bio-fluids using pl-CSA binding protein and an anti-pl-CSA antibody. Immunohistochemical staining of tissue chips was used as the gold standard control.

**Results:** The developed ELISA method was specific and sensitive (1.22 μg/ml). The pl-CSA content was significantly higher in lysates and supernatants of cancer cell lines than in those of normal cell lines, in plasma from mouse cancer models than in that from control mice, and in plasma from patients with oesophageal, cervical, ovarian, or lung cancer than in that from healthy controls. Similar to the tissue chip analysis, which showed a significant difference in pl-CSA positivity between cancer tissues and normal adjacent tissues, the plasma pl-CSA analysis had 100% sensitivity and specificity for differentiating oesophageal and lung cancer patients from healthy controls. Importantly, in oesophageal and lung cancer patients, the pl-CSA content was significantly higher in late-stage disease than in early-stage disease, and it dramatically decreased after surgical resection of the tumour.

**Conclusion:** These data indicate a direct link between plasma pl-CSA content and tumour presence, indicating that plasma pl-CSA may be a non-invasive biomarker with clinical applicability for the screening and surveillance of patients with multiple types of solid tumours.

## Introduction

Cancer remains a serious public health problem worldwide. According to reports from the International Agency for Research on Cancer (IARC), approximately 18.1 million new cancer cases and 9.6 million cancer deaths are detected annually worldwide 1. In China, approximately 4.3 million new cancer cases and 2.81 million cancer deaths occur every year 2. In clinical practice, the prognosis of cancer patients is associated with the pathological stage at the time of diagnosis. However, in over 70% of patients, cancer is detected and diagnosed at an advanced stage. Therefore, early screening and diagnosis are very important for prompt treatment and a good prognosis.

In the process of tumourigenesis, circulating tumour cells (CTCs), debris and secreted substances are shed from the primary tumour into peripheral blood and have potential for use in the early detection of cancer. Current cancer biomarkers in clinical use are primarily DNA 3, RNA 4, and protein 5. These types of biomarkers are useful as complementary tools for traditional pathological staging in cancer diagnostics and prognostics. However, their application in the early detection of a malignancy is limited by the relatively low sensitivity and specificity 6. Therefore, there is a need to discover new cancer biomarkers to improve screening and diagnostic efficiency.

It has been reported that glycosaminoglycans (GAGs), a type of polysaccharide, play a key role in tumourigenesis 7. Placental-like chondroitin sulfate (CS) A (pl-CSA) belongs to the GAGs family and serves as a receptor for *Plasmodium falciparum*-infected erythrocytes through the anchor protein VAR2CSA 8. Pl-CSA contains a typical GlcA-GalNAc (4S) disaccharide unit and shows relatively less variation than DNA, RNA and protein 9; it is exclusively synthesized in cancer and placental tissues and is associated with cancer progression 10. Studies have also confirmed that recombinant VAR2CSA (rVAR2) can specifically bind to pl-CSA in multiple cancer types 10 and can serve as a target to isolate CTCs 11. These properties of pl-CSA suggest that its content in tumour tissue and bio-fluids may be a promising biomarker for cancer screening and diagnosis. However, whether pl-CSA is released in bio-fluids as a cancer biomarker remains unclear.

In this study, circulating pl-CSA content was analysed through a new ELISA method using a pl-CSA binding peptide derived from VAR2CSA and an anti-pl-CSA antibody 12, 13. The pl-CSA content was measured in plasma from patients with different types of cancer. The results revealed that deregulated circulating pl-CSA content is a promising cancer biomarker with high specificity and sensitivity in distinguishing cancer patients from healthy controls.

## Materials and Methods

### Development of a novel ELISA kit for pl-CSA analysis

As specified in the supplementary methods, purified pl-CSA was obtained from HTR8 cells, and pl-CSA anti-serum was also collected from immunized mice. The pl-CSA binding peptide (pl-CSA-BP), a mini-peptide derived from VAR2CSA 14, 15, was synthesized (China Peptides Co., Ltd., Shanghai, China). The concentrations of capture protein (supplementary Table S1) and anti-pl-CSA antibody (supplementary Table S2) were optimized, and the specificity (supplementary Table S3), sensitivity and repeatability of the ELISA were determined.

**Figure 1:**
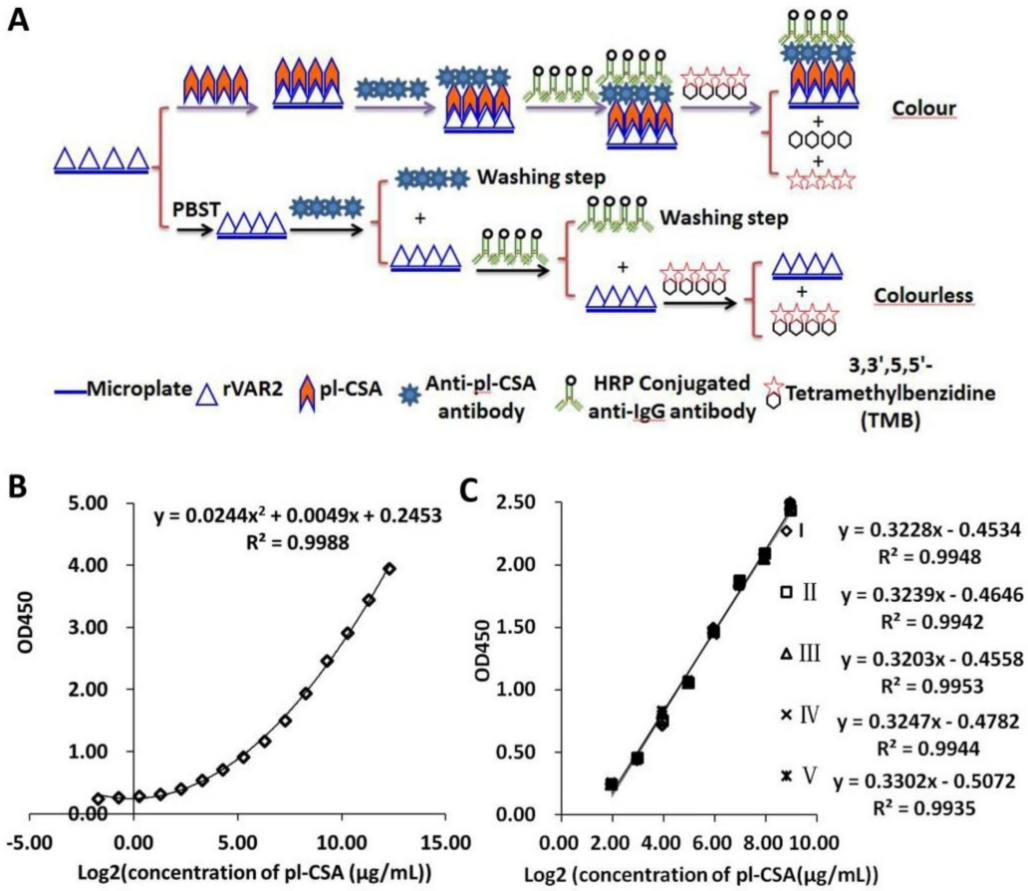
** Specificity and repeatability of the double-antibody sandwich ELISA. (A**) Assay sensitivity: the pl-CSA concentrations were 0.31 μg/ml, 0.61 μg/ml, 1.22 μg/ml, 2.44 μg/ml, 4.88 μg/ml, 9.77 μg/ml, 19.53 μg/ml, 39.06 μg/ml, 78.13 μg/ml, 156.25 μg/ml, 312.50 μg/ml, 625.00 μg/ml, 1250.00 μg/ml, 2500.00 μg/ml, and 5000.00 μg/ml. The lowest detectable concentration was 0.31 μg/ml. (**B**) Assay repeatability: The pl-CSA concentrations were 3.91 μg/ml, 7.81 μg/ml, 15.63 μg/ml, 31.25 μg/ml, 62.50 μg/ml, 125.00 μg/ml, 250.00 μg/ml, and 500.00 μg/ml. The content in each sample was measured 5 times.

The general ELISA protocol was as follows (schematic illustration, Figure 1A). First, pl-CSA-BP was dissolved in 50.00 mM carbonate buffer (pH 9.6) and coated on 96-microwell plates (200 μl/well) overnight at 4 °C. Second, the plate was incubated with 1% BSA (200 μl/well) for 2 h to block nonspecific reactions, with no washing steps. Third, the pl-CSA samples and standard samples (100 μl/well) were incubated in the plates for 2 h. Fourth, the anti-pl-CSA antibody was added, and the plates were incubated for 2 h. Fifth, horseradish peroxidase (HRP)-labelled goat anti-mouse IgG antibody (100 μl/well, 1:10,000 dilution) was added for 1 h, and peroxidase activity in the wells was detected by adding 100 μl of substrate solution (3,3',5,5'-tetramethylbenzidine (TMB)). After 15 min, the reaction was stopped with 2 M H_2_SO_4_ (50 μl/well), and the absorbance was recorded at 450 nm (OD450). The plate was placed at 37 °C for all incubation steps and washed three times (for 5 min each) with 0.1 M PBST (pH 7.2) after each incubation step. The same protocol was applied for all ELISAs. The reliability of the values was confirmed by P/N≥2.1.

### Quantitative determination of pl-CSA in bio-fluids from *in vitro* and *in vivo* samples

To explore whether pl-CSA is released into bio-fluids by cancer cells *in vitro*, cell culture supernatants (36 samples) and cell lysates (36 samples) were collected (supplementary methods). A total of 17 cell lines (supplementary Table S4) were obtained from American Type Tissue Collection (ATCC, Manassas, VA) from June 2010 to April 2016 and stored in liquid nitrogen in our laboratory. The human trophoblast cell line HTR8 (a kind gift from Professor Charles Graham, Department of Anatomy and Cell Biology, Queen's University, Kingston, ON, Canada) was maintained in our laboratory. All cell lines were authenticated using short tandem repeat (STR) analysis according to the ANSI standard (ANSI/ATCC ASN-0002-2011 Authentication of Cell Lines: Standardization of STR Profiling) by the ATCC Standards Development Organization (SDO) and tested negative for mycoplasma contamination. All authentications were carried out by Guangdong Hybribio Biotech Ltd. in July 2016.

To explore whether pl-CSA is released into the circulatory system *in vivo*, plasma samples were collected from mouse cancer models, including from choriocarcinoma BALB/c nude mouse models (10 samples), ovarian cancer models (10 samples) and negative controls (10 samples) used in previous studies 15,16. The pl-CSA content in cell samples and mouse plasma samples was quantified using the developed ELISA method.

### Measurement of circulating pl-CSA in patients with different types of cancer

For all patients, the diagnosis was pathologically confirmed by two independent pathologists. No patients received radiotherapy or chemotherapy prior to surgery. For case-control subjects, blood samples were taken once before operation from cancer patients and upon recruitment from controls. For pre- and post-surgery or pre- and post-chemotherapy plasma samples, venous blood samples were collected twice from cancer patients: one day before the operation and 3-7 days after surgery. For each sample, ~10 ml of whole blood was collected in an EDTA-containing tube and centrifuged at 5,000 rpm and 4 °C for 10 min. The supernatants were stored at -80 °C until ELISA analysis.

To validate the clinical significance of circulating pl-CSA, the pl-CSA concentration in plasma samples from patients with different types of cancer was quantified using the developed ELISA method; the samples were from 48 patients with oesophageal squamous cell carcinoma (ESCC) (66 samples), 7 with ovarian cancer (7 samples), 7 with cervical cancer (7 samples), 32 with non-small-cell lung cancer (NSCLC) (52 samples), and 44 healthy controls (44 samples) (supplementary Table S5).

### Immunohistochemical analysis of tissue chips

The pl-CSA content in oesophageal (cat No. ES1202; 10 × 12 1-mm holes/chip; 60 cases) and lung cancer (cat No. BC04118a; 10 × 10 1-mm holes/chip; 50 cases) tissues, as well as in normal adjacent tissues, was analysed through tissue chip immunohistochemistry by Suzhou Cancer Cell Biotechnology Co., Ltd. (supplementary Table S6). Briefly, the tissue chip sections were baked, deparaffinised and rehydrated; then, antigen retrieval was performed, and endogenous HRP was inactivated. The sections were incubated consecutively with biotinylated pl-CSA-BP, HRP-conjugated streptavidin, and diaminobenzidine (DAB, chromogen substrate) and counterstained with haematoxylin. Then, the sections were visualized using a microscope (BX53, Olympus Corporation, Tokyo, Japan), and the microphotographs were analysed using Image-Pro Plus 6.0 software. The percentage of positive cells with different staining intensities dictated the staining score: no positive cells, 0; ≤ 10% positive cells, 1; 10% < positive cells ≤ 50%, 2; 50% < positive cells ≤ 80%, 3; and ≥ 80% positive cells, 4.

### Statistical analysis

All statistical analyses were performed using the SAS statistical software package (version 9.4, SAS, Cary, NC). Continuous variables are presented as the mean ± SD. Differences between groups were compared using chi-square tests for categorical variables and Student's t test or the median test for continuous variables, where appropriate. The receiving operating characteristics (ROC) curve was used to investigate the discriminatory potential of plasma pl-CSA content. *P* values of <0.05 were considered to indicate statistical significance.

## Results

### The newly developed ELISA method showed high specificity and sensitivity

The optimized concentration of pl-CSA-BP for ELISA was determined to be 25.00 μg/ml. Purified pl-CSA at concentrations greater than 5.00 mg/ml was obtained through rVAR2 16 affinity chromategraphy, dialysis and vacuum drying. Approximately 5 ml of anti-pl-CSA serum was collected from BALB/c mice immunized with purified pl-CSA (25.00 mg/kg body weight) at total of five times at an interval of one week. The optimum dilution ratio of 1:1,000 for anti-pl-CSA serum was confirmed (supplementary Table S2).

The specificity of the ELISA was confirmed using the GAG similarity of CSB and CSC, which had OD450 values similar to that of the negative control, P/N= ~1.00 (supplementary Table S3), suggesting that the ELISA had relatively high specificity. The log_2_-transformed pl-CSA concentration (ranging from 1.22 μg/ml to 625.00 μg/ml) showed a linear relationship with OD450. When the pl-CSA concentration was below 1.22 μg/ml or above 625.00 μg/ml, a colour change was observed, but the relationship was not linear (Figure 1B). Under the specified conditions, the sensitivity of this ELISA kit for detecting pl-CSA was 1.22 μg/ml. The regression coefficients and correlation coefficients were similar in five experiments, with a variable coefficient of less than 1% between the pl-CSA concentration (ranging from 3.91 μg/ml to 500.00 μg/ml) and the OD450 value (Figure 1C), indicating good repeatability of the ELISA kit.

### Pl-CSA was detected in bio-fluids of cancer cells but not of normal cells

**Figure 2:**
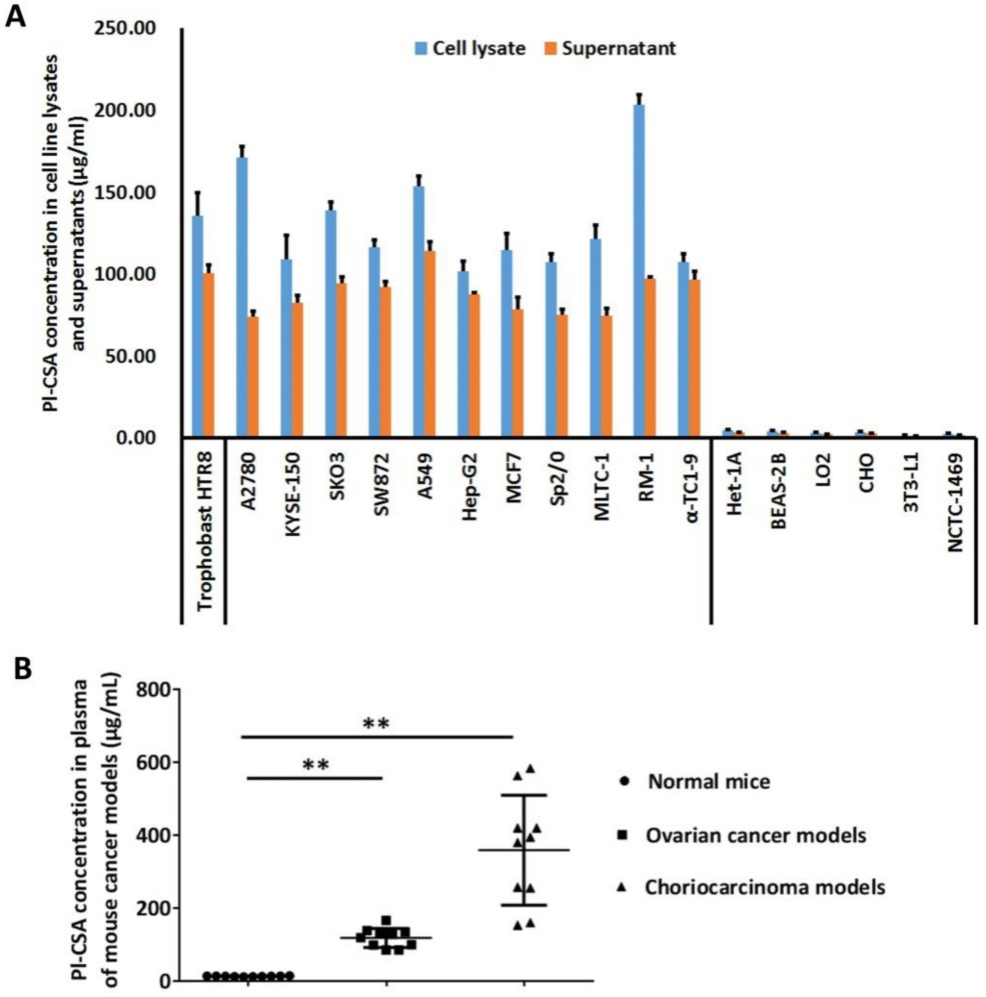
** Pl-CSA content was increased in the supernatants and lysates of cancer cells and in the plasma from mouse cancer models. (A)** The cell lysates and cell culture supernatants from fifteen cell lines were collected and analysed using the double-antibody sandwich ELISA. The evaluated cell lines included six human cancer cell lines and four mouse cancer cell lines, as well as two normal human cell lines and two normal mouse cell lines as negative controls and the HTR8 cell line as a positive control.** (B)** The plasma concentration of pl-CSA was significantly higher in mouse cancer models than in control mice.

The pl-CSA content in the culture supernatants and lysates of 18 cell lines was determined using the developed ELISA method. The pl-CSA concentration was above 50.00 μg/ml in the lysates of 11 cell lines of different cancer types, including A2780, KYSE-150, SKO3, SW872, A549, Hep-G2, MCF7, Sp2/0, MLTC-1, RM-1, and αTC1-9 cells; the HTR8 trophoblast cell line served as a positive control. After 5-fold concentration, the pl-CSA concentration in the cell culture supernatants was above 50.00 μg/ml. However, the pl-CSA content in the lysates and supernatants of the normal cell lines, including Het-1A, BEAS-2B, LO2, CHO, 3T3-L1 and NCTC-1469 cells, was under the detectable limit (Figure 2A). These results suggest that pl-CSA is released into bio-fluids by cancer cells.

### The plasma pl-CSA content was higher in mouse cancer models than in normal control mice

The pl-CSA concentration in plasma samples from mouse choriocarcinoma and ovarian cancer models was analysed. The results showed that the pl-CSA concentration was above 100.00 μg/ml in all plasma samples from both mouse cancer models and was significantly higher in the mouse cancer models than in the normal control mice (all below 10.00 μg/ml) (Figure 2B). These results suggest that pl-CSA is released into the circulatory system *in vivo*.

### The plasma pl-CSA content was higher in patients with ovarian, cervical, oesophageal or lung cancer than in healthy controls

**Figure 3:**
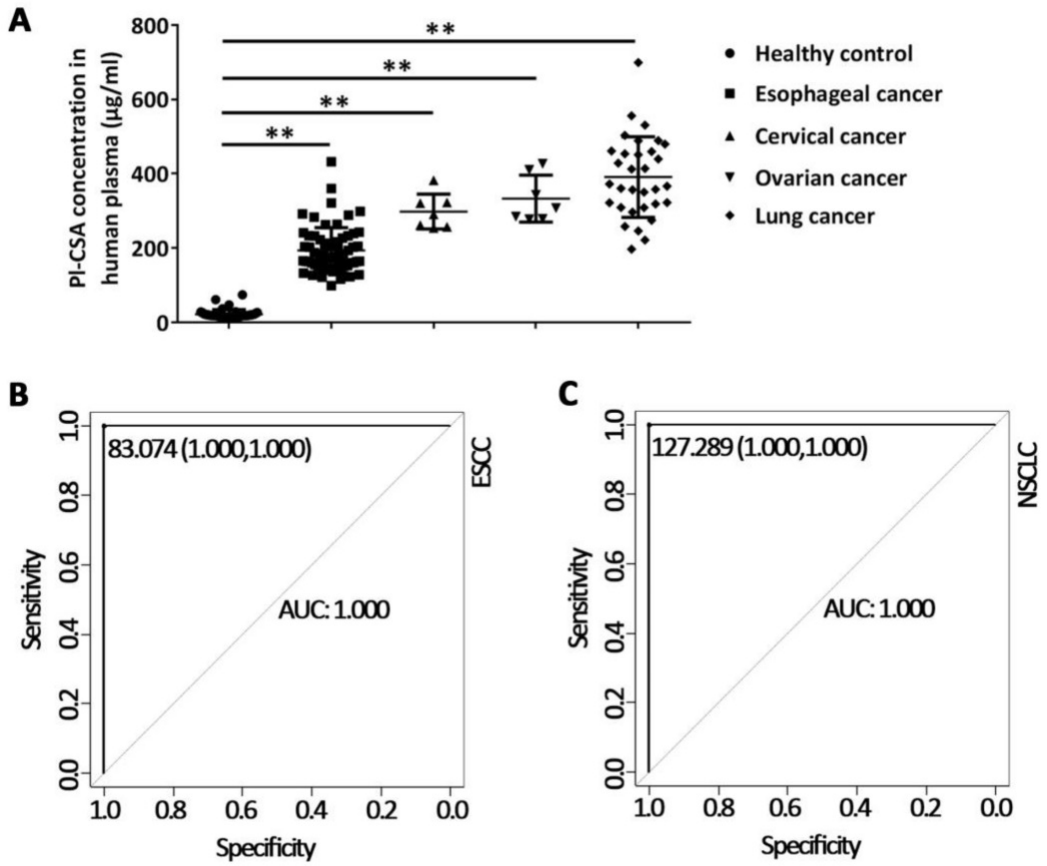
** Plasma Pl-CSA content was higher in cancer patients than in healthy controls. (A)** The plasma pl-CSA content was significantly higher in patients with oesophageal cancer, ovarian cancer, cervical cancer or lung cancer than in healthy controls. **(B)** ROC curve analysis of plasma pl-CSA for differentiating oesophageal cancer patients from healthy controls. **(C)** Sensitivity and specificity of plasma pl-CSA content for distinguishing lung cancer patients from healthy controls.

To investigate whether the circulating pl-CSA content can distinguish cancer patients from healthy controls, the pl-CSA concentration was determined in plasma samples from patients with ovarian, cervical, oesophageal or lung cancer and in those from healthy controls. The plasma pl-CSA concentration was above 100.00 μg/ml in samples from the patients with cancer and was significantly higher in cancer patients than in healthy controls, who had a concentration below 60.00 μg/ml (Figure 3A). The discriminatory capability of plasma pl-CSA content was evaluated through receiver operating characteristic (ROC) curve analysis, which revealed 100% sensitivity and specificity for ESCC and NSCLC (Figure 3B and C). These results indicate that higher plasma pl-CSA levels might be a sensitive biomarker of solid tumours.

**Figure 4:**
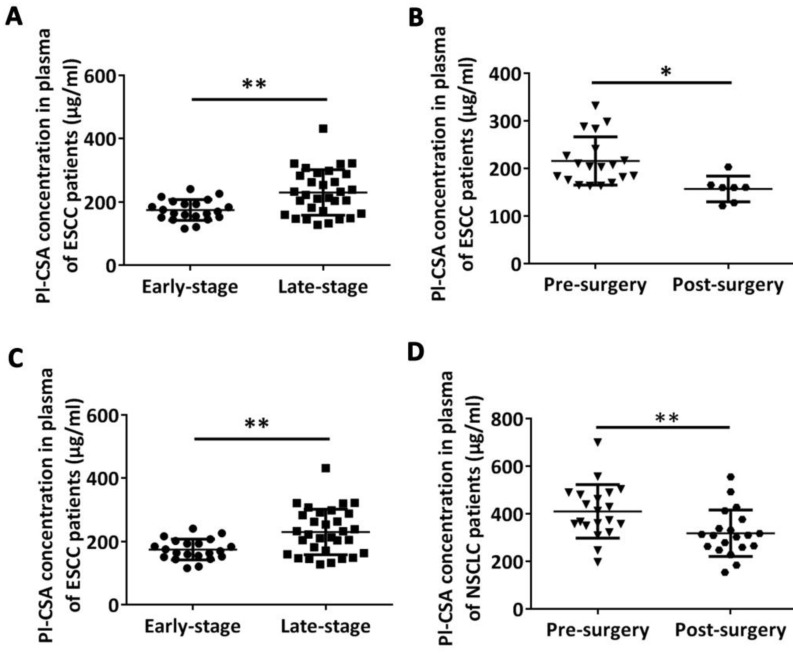
** Plasma pl-CSA context was higher in late-stage patients and was significantly decreased after surgical tumour removal in patients with oesophageal or lung cancer. (A)** The plasma concentration of pl-CSA was significantly higher in late-stage patients with oesophageal cancer than in early-stage patients. **(B)** The plasma content of pl-CSA in oesophageal cancer patients was lower after surgery than before surgery. **(C)** The plasma concentration of pl-CSA in late-stage patients with lung cancer was significantly higher than that in early-stage patients. **(D)** The plasma content of pl-CSA in patients with lung cancer was lower after surgery than before surgery.

To assess whether the plasma pl-CSA content is correlated with the clinical stage of cancer, we compared pl-CSA levels in patients with early-stage or late-stage ESCC or NSCLC. The results showed that the mean pl-CSA content was significantly higher in patients with late-stage oesophageal cancer (230.40 ± 89.13 μg/ml) than in those with early-stage disease (175.00 ± 21.67 μg/ml) (Figure 4A) and was significantly higher in patients with late-stage NSCLC (476.6 ± 110.00 μg/ml) than in those with early-stage disease (366.40 ± 40.27 μg/ml) (Figure 4C). Taken together, these data provide evidence that pl-CSA levels are positively correlated with cancer progression.

**Figure 5:**
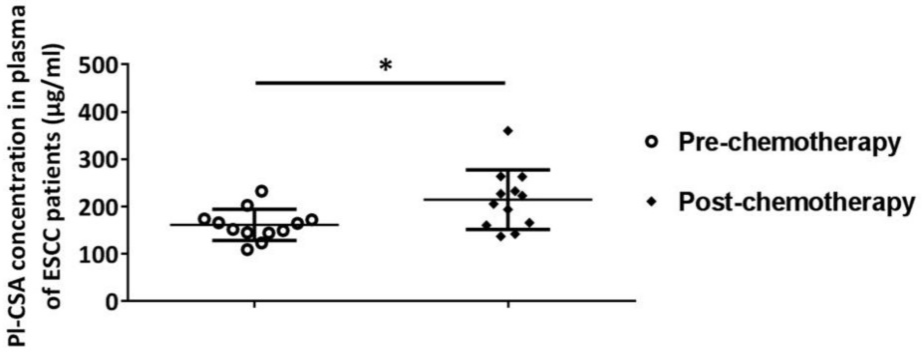
** Plasma pl-CSA content was significantly increased after chemotherapy in patients with oesophageal cancer.** The plasma concentration of pl-CSA in pre-chemotherapy patients with oesophageal cancer was significantly lower than that in post-chemotherapy patients.

To evaluate whether changes in plasma pl-CSA are associated with clinical treatments, the plasma pl-CSA content in patients with oesophageal and NSCLC was compared before and after surgical resection. The plasma pl-CSA content was significantly lower after the surgical resection of an oesophageal tumour (157.10 ± 20.31 μg/ml) than before surgery (216.10 ± 58.94 μg/ml) (Figure 4B). Likewise, the surgical removal of lung cancer tissue significantly decreased the pl-CSA content, as evidenced by the comparison between post-operative plasma samples (318.20 ±33.28 μg/ml) and pre-operative plasma samples (410 ± 91.90 μg/ml) (Figure 4D). Interestingly, in the oesophageal patients, the plasma pl-CSA content was significantly higher after chemotherapy than before chemotherapy (Figure 5). These results suggest that a high pl-CSA content might be associated with the number of cancer cells. However, the significant increase in plasma pl-CSA content in patients with ESCC after chemotherapy suggests that pl-CSA may be a real-time marker for monitoring the effect of chemotherapy.

### Pl-CSA was detectable in cancer tissues but not in normal adjacent tissues

**Figure 6:**
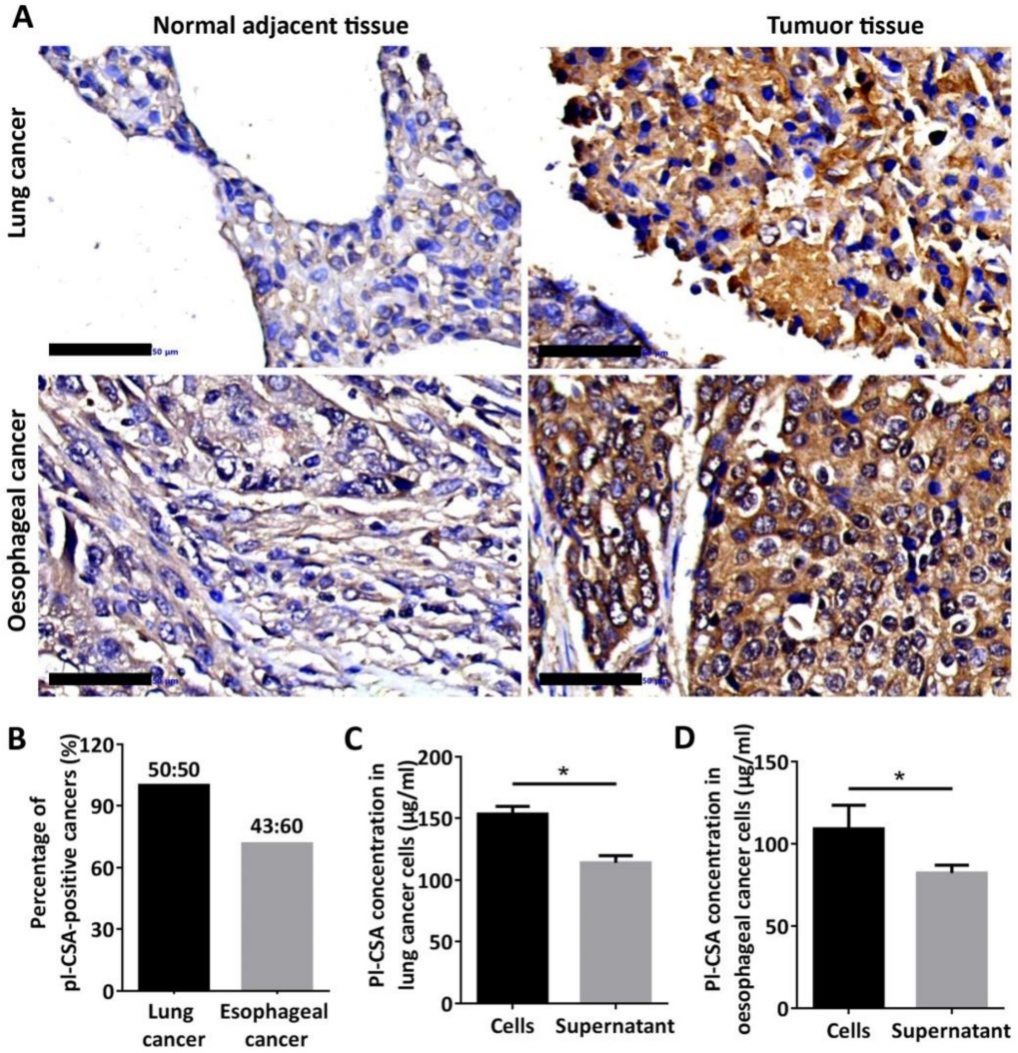
** Pl-CSA was synthesized in cancer tissue and cancer cell lines and released in bio-fluids. (A)** Pl-CSA was detectable by immunohistochemistry in oesophageal and lung cancer tissue but not in normal adjacent tissue (scale bar: 50 μm). **(B)** The analysis of pl-CSA-positive cases indicated 72% and 100% positivity in oesophageal and lung cancer tissues, respectively, compared with normal adjacent tissue. **(C)** A small amount of pl-CSA was released into the supernatant by the lung cancer cell lines, and the pl-CSA content was significantly higher in the lysates than in the supernatants. **(D)** A small amount of pl-CSA was released into the supernatant by the oesophageal cancer cell lines, and the pl-CSA content in the lysates was significantly higher than that in the supernatants.

Through immunohistochemistry analysis of tissue chips, pl-CSA was detected in cancer tissues, primarily in the cytoplasm, but not in normal adjacent tissues (Figure 6A). Compared with the normal adjacent tissues, approximately 72% and 100% of the oesophageal and lung cancer tissues, respectively, were positive for pl-CSA (Figure 6B). This marker was also detected in lysates and supernatants of cultured oesophageal and lung cancer cells, and the pl-CSA content was significantly higher in lysates than in supernatants (Figure 6C and D).

## Discussion

To the best of our knowledge, this study is the first to investigate the role of circulating pl-CSA as a cancer biomarker. We found that the pl-CSA content was increased in supernatants of multiple cancer cells, plasma from mouse cancer models, and plasma from patients with cancer. A higher plasma pl-CSA content was correlated with cancer TNM stage. In addition, surgical removal of the tumour significantly reduced the plasma pl-CSA content. These findings suggest that circulating pl-CSA may be a promising biomarker for cancer screening and diagnosis.

GAGs are one of the most important components of the extracellular matrix (ECM). As a subunit of GAGs, CS is covalently bound to CS proteoglycans (CSPGs) in many tissues and cancer cells 17, 18. CSPGs can regulate key cellular processes, including proliferation, apoptosis, migration, adhesion, angiogenesis, and invasion, as well as ECM assembly via their highly negatively charged CS side chain 19. Many cancer cells express a distinct CS subtype that is normally restricted to trophoblastic cells in the placenta, indicating their functional importance in cancer pathology 10. These placental-type CSs are synthesized by tumour cells and tumour-infiltrating stromal cells across multiple types of cancer, again suggesting their functional importance in cancer pathology. In support of this hypothesis, a recent study showed that placental-type CS is required for cancer cellular processes *in vitro* and *in vivo* by modifying the integrin signalling pathway, suggesting that placental CS may be a candidate therapeutic target 20. Indeed, the quantitative analysis of GAG subunits in circulation has been proposed as a new method for identifying biomarkers that facilitate diagnosis, predict clinical severity and prognosis, and enable treatment monitoring and disease screening 21, 22.

Pl-CSA is expressed in many cancer tissues and associated with disease severity 10. However, whether the pl-CSA content in bio-fluids can be used as a biomarker for cancer screening and diagnosis has not been validated. In this study, the pl-CSA content was significantly higher in the supernatants of cancer cells than in the supernatants of normal cells and in sera from animal cancer models or patients with various types of cancer than in healthy controls. Our data indicate that pl-CSA can be released into bio-fluids, and the pl-CSA content in bio-fluids may be a target for the development of a new biomarker for cancer diagnosis or prognosis. In addition, circulating pl-CSA content was higher in late-stage patients than in early-stage patients, suggesting that circulating pl-CSA concentration is associated with disease severity and may be used for cancer grading and staging. Moreover, our data show that the surgical removal of tumours can significantly reduce plasma pl-CSA concentrations, as shown by comparisons with pre-operative plasma samples, indicating that tumour tissue is the specific origin of the elevated plasma pl-CSA. In contrast, we observed a significant increase in plasma pl-CSA content in patients with ESCC after chemotherapy, suggesting that pl-CSA may be a real-time indicator of the effect of chemotherapy. The mechanisms by which chemotherapy increases pl-CSA content are unclear. Elevated pl-CSA content may be partly due to the damaging effect of chemotherapeutic agents on cancer cells since pl-CSA was abundant on cancer cells and killing cancer cells may prompt the release of more pl-CSA into circulation. Further clinical validation in larger prospective trials is needed to determine the changes in plasma pl-CSA content in response to chemotherapy.

No specific assays for the quantification of circulating pl-CSA had been developed. In the present study, we developed a novel ELISA to quantify pl-CSA content in bio-fluids. To increase the detection rate, we designed and generated a polyclonal antibody kit targeting multiple epitopes of pl-CSA; in this kit, pl-CSA is the antigen, pl-CSA-BP (VAR2CSA) is the capture protein, and the anti-pl-CSA antibody is the detection antibody. An ELISA is preferentially used to map epitopes of different monoclonal antibodies that have been generated against a single antigen 23. Antigen detection by capture proteins and the detection of polyclonal antibodies improve the binding specificity and sensitivity of the assay 24. In the present study, both pl-CSA-BP and pl-CSA antibodies selectively bound pl-CSA to improve the binding specificity. As the detection antibody is a polyclonal antibody, it can bind to multiple epitopes of the antigen, thus reducing the missed detection rate. This method increases the sensitivity of the assay, and the antigen-antibody reaction and enzyme-catalysed reaction ensure the specificity, which was verified with the homologous compounds CSB and CSC in our experiments. In the present study, we used the optimized ELISA to detect the plasma pl-CSA concentration in patients with ESCC and NSCLC. The results showed that this ELISA had 100% specificity and 100% specificity in distinguishing ESCC and NSCLC patients from healthy controls.

In summary, our findings suggest that a higher plasma pl-CSA content might be a novel, sensitive diagnostic biomarker of cancer. Our study provides evidence that a higher plasma pl-CSA content may also by a biomarker of cancer severity and the response to chemotherapy. Further studies are required to validate our findings in a large number of samples.

## Supplementary Material

Supplementary tables.

## Abbreviations

CS: chondroitin sulfate
pl-CSA: placental-like chondroitin sulfate A
pl-CSA-BP: pl-CSA binding peptide
GAGs: glycosaminoglycans
ESCC: oesophageal squamous cell carcinoma
NSCLC: non-small-cell lung carcinoma
CTCs: circulating tumour cells
IHC: immunohistochemistry
ELISA: enzyme-linked immunosorbent assay
