# Supplementary material for: Screening and surveillance of multiple solid tumours using plasma placental-like chondroitin sulfate A (pl-CSA)
Source: Int J Med Sci. 2020 Jan 1;17(2):161–9. doi: 10.7150/ijms.39444 (PMC6990888; doi:10.7150/ijms.39444)
Supplement: Supplementary file 1 — Supplementary tables. [file ijmsv17p0161s1.pdf]

Supplementary methods

Pl-CSA purification

As in our previous study, the affinity protein rVAR2 was used for the purification of pl-CSA. The purified rVAR2 was quantified using an Easy II Protein Quantitative kit (BCA) (Cat. No. DQ111-01, Transgen Biotech Co., Ltd., Beijing, China). The 4% N-hydroxysuccinimide (NHS)-activated agarose reacted with 1-ethyl-3-(3-dimethylamino) propyl carbodiimide (EDC) in 100 mM 4-morpholinoethanesulfonic acid (MES) solution (pH 5.5) (Cat. No. SA039500, Smart Lifesciences, Changzhou, China) at room temperature (RT) for 2 h. Then, the rVAR2 protein was coupled to activated agarose at a concentration of 1 mg/g at RT for 24 h. The uncoupled rVAR2 was removed by washing with PBS (pH 7.2). An affinity column was filled with the prepared agarose for pl-CSA purification.

The crude pl-CSA sample was obtained from HTR8 cells using an enzyme microwave-assisted method with lysis buffer (pH 8.0) containing 1 IU/ml RNase, 2 IU/ml DNase, and 1 μg/ml protease K. The sample was treated with microwaves at 37 °C for 30 min and incubated at 4 °C for 16 h; then, enzyme inactivation was performed at 85 °C for 2 min, and the sample was passed through a 0.45-μm filter. The purified pl-CSA was collected using an rVAR2 affinity column at a ratio of 1 ml of crude pl-CSA per 1 ml of column, in which PBS (pH 7.2) was used as a washing solution and 0.10 M glycine buffer (pH 3.0) served as the elution buffer. Then, the purified pl-CSA was deionized, freeze-dried and stored at -80 °C for the following experiments.

Mouse immunization

The pl-CSA vaccine was produced through emulsification with Freund’s complete adjuvant or Freund’s incomplete adjuvant. Ten BALB/c mice were vaccinated subcutaneously with the pl-CSA vaccine at an interval of one week. The mice were immunized with vaccine in Freund’s complete adjuvant in the first and second weeks, with vaccine in Freund’s incomplete adjuvant in the third and fourth weeks, and with pl-CSA alone in the last week. The serum of immunized mice, which contained the anti-pl-CSA antibody, was collected. The pl-CSA antiserum was mixed and stored at -80 °C for ELISA analysis.

Capture protein preparation

To ascertain the appropriate concentration of pl-CSA-BP for coating the ELISA plates, a checkerboard titration was applied using the protocols described above (Table S1). In the first step,

the pl-CSA-BP was serially diluted 2-fold to generate a concentration gradient (range, 3.13 μg/ml to 100.00 μg/ml). In the third step, the pl-CSA was serially diluted 10-fold to generate standard samples for the concentration gradient. In the fourth step, the anti-pl-CSA antibody was tested at a 1:1,000 dilution.

Optimization of the anti-pl-CSA antibody dilution ratio

The optimum anti-pl-CSA antibody dilution ratio was determined by checkerboard titration based on the above-described protocols (Table S2). In the third step, the pl-CSA standards were generated by serial 2-fold dilution, each with twelve replicates. Subsequently, the pl-CSA antiserum and mouse control serum were serially diluted 10-fold, with 3 replicates of each dilution.

Determination of the specificity, sensitivity and repeatability of the ELISA kit

Based on the above-described protocols, in the third step, serial 2-fold dilutions of purified pl-CSA were used as standards to determine the sensitivity (range, 0.31 μg/ml to 5,000.00 μg/ml) and repeatability (range, 3.91 μg/ml to 500.00 μg/ml). The concentration of purified pl-CSA was $\log_2$-transformed, and the resulting values exhibited a linear relationship with the OD450. The assay was repeated more than three times to determine the sensitivity and repeatability. CSB (500.00 μg/ml) and CSC (500.00 μg/ml) were also detected to determine the specificity. The reliability was confirmed according to P/N≥2.1.

Cell culture

Most cell lines were obtained from American Type Tissue Collection (ATCC, Manassas, USA) and maintained in our laboratory. Cell lines were cultured in appropriate media supplemented with 10% foetal bovine serum (FBS), 100.00 IU/ml penicillin, and 100.00 μg/ml streptomycin. The HTR8 cell line (a kind gift from Professor Charles Graham, Department of Anatomy and Cell Biology, Queen's University, Kingston, ON, Canada) was used as a positive control, and the 11 cancer cell lines and 6 normal cell lines are shown in Table S4. When the cells reached 90% confluence, they were washed twice with PBS and cultured in FBS-free media for 48 h. Then, the culture supernatants were collected and condensed 5 times, and the cells were resuspended at approximately 10^6 cells/ml and lysed in lysis buffer for pl-CSA detection. Additionally, trophoblastic HTR8 cells were expanded for pl-CSA separation and purification.

Supplementary Table 1. The appropriate concentration of pl-CSA binding peptide (pl-CSA-BP) was identified through checkerboard titration.

OD450		pl-CSA-BP (μg/ml)					
		100.000	50.000	25.000	12.500	6.250	3.125
pl-CSA (μg/ml)	5,000.000	3.297	3.311	3.387	3.151	2.987	2.566
	500.000	1.944	1.959	1.988	1.979	1.941	1.918
	50.000	1.102	1.013	1.091	1.161	1.079	1.081
	5.000	0.401	0.402	0.415	0.409	0.403	0.415

Note: An assigned dilution (1:1,000) of the anti-pl-CSA antibody was used in this assay.

Supplementary Table 2. The optimum anti-pl-CSA antibody dilution ratio was determined through checkerboard titration.

OD450		pl-CSA (μg/ml)							
		3.906	7.813	15.625	31.250	62.500	125.000	250.000	500.000
1:100	Positive serum	0.659	1.018	1.254	1.491	1.850	2.067	2.554	2.573
	Negative serum	0.258	0.273	0.278	0.272	0.292	0.282	0.307	0.303
	P/N	2.558	3.726	4.512	5.488	6.334	7.344	8.326	8.485
1:1,000	Positive serum	0.247	0.442	0.717	1.064	1.495	1.835	2.084	2.497
	Negative serum	0.086	0.088	0.089	0.099	0.101	0.099	0.101	0.095
	P/N	2.873	5.025	8.107	10.751	14.876	18.534	20.735	26.419
1:10,000	Positive serum	0.221	0.229	0.399	0.552	0.775	0.954	1.082	1.266
	Negative serum	0.095	0.092	0.096	0.109	0.110	0.109	0.110	0.103
	P/N	2.331	2.495	4.156	5.086	7.020	8.781	9.801	12.267
1:100,000	Positive serum	0.206	0.225	0.299	0.414	0.581	0.715	0.812	0.950
	Negative serum	0.091	0.095	0.092	0.104	0.106	0.104	0.106	0.099
	P/N	2.274	2.373	3.260	3.968	5.473	6.851	7.641	9.591

Note: The 96-microwell plate was coated with 25 μg/ml pl-CSA-BP (200 μl/well) in 50 mM carbonate buffer (pH 9.6). P/N ≥ 2.1 was used to indicate reliable values.

Supplementary Table 3. The specificity of the ELISA for pl-CSA using CSB and CSC.

OD450	Negative control	CSB (500 μg/ml)	CSC (500 μg/ml)	pl-CSA (125 μg/ml)
1	0.102	0.121	0.116	1.699
2	0.106	0.107	0.108	1.732
3	0.110	0.134	0.108	1.765
4	0.104	0.098	0.112	1.753

Note: PBST was used as a negative control.

Supplementary Table 4. The cell lines used in this study to evaluate cell culture supernatants and lysates by the developed ELISA.

Serial No.	Cell line	Category	Group	Samples
1	HTR8	Trophoblast	Positive control	Two cell lysate samples and 2 supernatant samples for each cell line
2	A2780	Ovarian cancer	Cancer cell lines	
3	KYSE-150	Oesophageal cancer		
4	SKOV3	Ovarian cancer		
5	SW872	Liposarcoma		
6	A549	Lung adenocarcinoma		
7	Hep-G2	Hepatocellular cancer		
8	MCF7	Breast cancer		
9	Sp2/0	Myeloma		
10	MLTC-1	Leydig cancer		
11	RM-1	Prostate cancer		
12	αTC1-9	Pancreatic cancer		
13	Het-1A	Oesophageal epithelial	Normal cell lines	
14	BEAS-2B	Lung epithelial		
15	LO2	Hepatocyte		
16	CHO	Ovarian epithelial		
17	3T3-L1	Embryonic fibroblast		
18	NCTC-1469	Liver epithelial		

Supplementary Table 5. ESCC samples were collected from the Department of Oncology of Wuzhou People's Hospital (Wuzhou, China). Ovarian and cervical cancer samples were collected from Nanshan Affiliated Hospital of Shenzhen University (Shenzhen, China). NSCLC samples were obtained from patients who underwent surgical resection at the Department of Thoracic Surgery of The First Affiliated Hospital of Shenzhen University School of Medicine (Shenzhen, China). The mean age of the ESCC patients (8 females and 40 males) was 62 years. Of the oesophageal cancer patients, 7 were treated with surgery, and 12 were treated with chemotherapy. Samples were collected from the same patients with oesophageal cancer before and after surgery or chemotherapy. The malignancies were early-stage in 21 patients and late-stage in 27 patients. The mean age of the ovarian cancer patients was 60 years, and 7 patients had early-stage disease. The mean age of the cervical cancer patients was 62 years, and 7 patients had early-stage disease. The mean age of the NSCLC patients (19 females and 13 males) was 57 years, and 20 of these patients underwent surgery. Samples were collected from the same patients with NSCLC before and after surgery. The malignancies were early-stage in 24 patients and late-stage in 8 patients. The mean age of the healthy controls (21 females and 23 males) was 59 years. ELISAs were performed according to the abovementioned protocols.

Cancer samples	Mean age	Females	Males	Pre- and post-surgery	Pre- and post-chemotherapy	Early-stage	Late-stage
Oesophageal cancer	62	8	40	7	12	21	27
Ovarian cancer	60	7	0	0	0	7	0
Cervical cancer	62	7	0	0	0	7	0
Lung cancer	57	19	13	20	0	24	8
Healthy controls	59	21	23	/	/	/	/

Supplementary Table 6. Tissue chips obtained from Suzhou Cancer Cell Biotechnology Co., Ltd., included oesophageal cancer (cat No. ES1202; 10 × 12 1-mm holes/chip; 60 cases), lung cancer (cat No. BC04118a; 10 × 10 1-mm holes/chip; 50 cases), and corresponding normal adjacent tissue (NAT).

Cat. No.	Cancer tissue	Mean age	Females	Males	Early-stage	Late-stage
ES1202	Oesophageal cancer	57	34	26	55	5
BC04118a	Lung cancer	57	12	38	43	7
